# Weighted spectral correlation angle target detection method for land-based hyperspectral imaging

**DOI:** 10.1007/s12200-023-00100-4

**Published:** 2023-12-11

**Authors:** Qianghui Wang, Bing Zhou, Wenshen Hua, Jiaju Ying, Xun Liu, Yue Cheng

**Affiliations:** 1 Army Engineering University of PLA, Shijiazhuang, 050000 China; 2 Army Engineering University of PLA, Nanjing, 210000 China

**Keywords:** Hyperspectral image, Spectral uncertain feature, Target detection, Land-based imaging condition, Weighted spectral correlation angle (WSCA)

## Abstract

In land-based spectral imaging, the spectra of ground objects are inevitably affected by the imaging conditions (weather conditions, atmospheric conditions, light conditions, zenith and azimuth angle conditions) and spatial distribution of targets, leading to uncertainties featured by “same object different spectrum”. That is, the spectrum of a ground object may change within a certain range under different imaging conditions. Traditional target detection (TD) methods are mainly based on similarity measurements and do not fully account for the spectral uncertainties. These detection methods are prone to false detections or missed detections. Therefore, reducing the impact of spectral uncertainties on TD is an important research topic in hyperspectral imaging. In this paper, we first review traditional TD methods and compare their principles and characteristics. It is found that the spectral correlation angle (SCA) method has good adaptability in land-based imaging. The shortcoming of the SCA method that it cannot reflect the local spectrum characteristics, is also analyzed. As the effect of spectral uncertainties cannot be completely overcome by the SCA method, a new similarity measurement method, the weighted spectral correlation angle (WSCA) method, is proposed. It can reduce the influence of spectral uncertainties on TD by increasing the weight of particular bands. Finally, we use two sets of experiments to analyze the effect of the WSCA method on TD. Its performance in overcoming spectral uncertainties caused by variations in imaging conditions or uneven spatial distributions of targets is tested. The results show that the WSCA method can effectively reduce the influence of spectral uncertainties and obtain a good detection result.

**Graphical Abstract:**

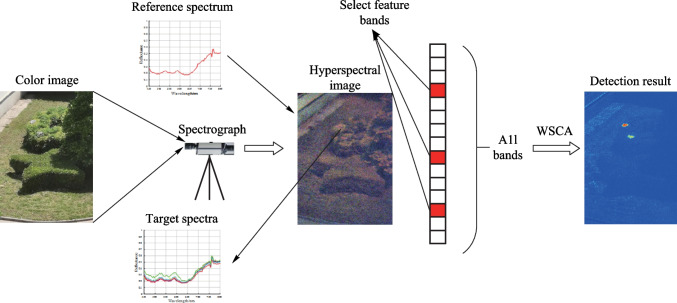

## Introduction

Before the advent of spectral imaging, the images obtained by traditional imaging technology were usually grayscale or color images. In those cases, the amount of information is relatively limited, and processing or analysis is mostly based on spatial information such as grayscale and texture distribution. Therefore, it can be impossible to distinguish the categories of ground objects from images [1]. With the development of imaging techniques, spectral imaging, which integrates spectral information into image data on the basis of traditional optical imaging techniques, has aroused much interest. It solves the problems of “only the image without spectrum” or “only the spectrum without image” with traditional optical imaging technology and makes it possible to analyze the categories of objects in an image [2, 3].

Hyperspectral imaging technology is an advanced data acquisition technology that records the spatial and spectral information of ground objects through an imaging spectrometer to obtain three-dimensional hyperspectral images (HSIs) [4, 5]. Its spectral resolution is usually below 10 nm, and the number of spectral bands can reach hundreds. It can obtain diagnostic spectral characteristics of ground objects and has wide applications in target detection (TD) [6], spectral decomposition [7], image classification [8], etc. In hyperspectral imaging, TD, that is obtaining the position of a desired target, can be realized by using the spatial and spectral information in the hyperspectral data. HSIs have the characteristics of high spectral resolution and numerous bands. They have great advantages in the field of TD [2, 9]. TD is often based on similarity measurements. It establishes a similarity relation between the test spectrum vector (the spectral vector of unknown pixels in the hyperspectral image) and the reference spectrum vector (the spectral vector of the known target). Then, the target with greater similarity to the reference spectrum can be identified [10].

In the ideal case, the category and spectrum of the target have a one-to-one correspondence; that is, the spectra of the same type of targets are the same, but the spectra of different types of targets are quite different. However, it is usually not the case in practice. Especially in land-based imaging, the spectral uncertainties of targets can be very significant, usually manifested as “same object different spectrum”. On one hand, the spectral curves of targets show uncertainties when imaging conditions change. On the other hand, the spatial distribution of targets is usually not uniform or regular, and obvious uncertainties exist when taking the whole large-area average spectrum as the spectrum of targets. These factors decrease the TD accuracy, which is determined by the similarity between the test spectrum vector and the reference spectrum vector. Therefore, it is necessary to reduce the effect of spectral uncertainties in land-based conditions.

The following sections of this paper are arranged as follows. Section 2 summarizes the principles of several traditional TD methods and analyzes the strengths and weaknesses of each method. Section 3 gives the principle and derivation process of the weighted spectral correlation angle (WSCA) method. Section 4 experimentally demonstrates the effectiveness of the WSCA method in reducing the spectral uncertainties. Section 5 is conclusion.

## Analysis of traditional TD methods

The basic form of TD in the hyperspectral image is a binary hypothesis testing problem, formulated as in Eq. (1):

1 $$D({\varvec{x}},{\varvec{y}}) = \left\{ {\begin{array}{*{20}c} { \ge \eta ,} & {\text{target,}} \\ { < \eta ,} & {\text{background,}} \\ \end{array} } \right.$$

where ${\varvec{x}} = [{\varvec{x}}_{1} ,{\varvec{x}}_{2} ,...,{\varvec{x}}_{n} ]$ represents the known reference spectrum vector. $\user2{y = }[{\varvec{y}}_{1} ,{\varvec{y}}_{2} ,...,{\varvec{y}}_{n} ]$ represents the unknown test spectrum vector. ${\varvec{x}}_{i}$ and ${\varvec{y}}_{i}$ represent the *i-*th value of spectrum vector $\varvec{x}$ and ${\varvec{y}}$ in the *i*-th band, respectively. $n$ represents the number of spectral bands. $\eta$ represents the threshold. $D({\varvec{x}},{\varvec{y}})$ represents the detection function. The TD methods work by comparing the known reference spectrum vector with the unknown test spectrum vector in a hyperspectral image. The pixels with high similarity are regarded as the target pixels and the pixels with low similarity are regarded as the background pixels. In general, TD methods can be categorized into projection-based, distance-based, information-based, and statistics-based methods. In addition, the constrained energy minimization (CEM) method is also a traditional TD method [11]. It is widely used in the field of TD. The idea is to enhance the information in the direction of interest and suppress the information in the direction of no interest, thus highlighting the target. The following is a brief introduction to the above mentioned traditional TD methods.

### Projection-based TD methods

Projection-based TD methods mainly exploit the shape differences between the reference spectrum vector and the test spectrum vector. This type of method is insensitive to differences in the spectral amplitude, which includes the spectral angle metric (SAM) [12] method, the spectral angle cosine (SAC) method, the spectral gradient angle (SGA) method, the normalized spectral gradient angle (NSGA) method, the kernel spectral angle (KSA) method [13] and orthogonal projection divergence (OPD) method [14]. Among them, The SAM, SAC, SGA, and NSGA methods are the most typical.

The SAM method evaluates the shape differences between spectral vectors by measuring the inclusive angle between the reference spectrum vector and the test spectrum vector. The SAM is defined as in Eq. (2):

2 $${\text{SAM}} \left( {{\varvec{x}},{\varvec{y}}} \right) = \cos^{ - 1} \frac{{{\varvec{xy}}^{\text{T}} }}{{\left[ {\left( {{\varvec{xx}}^{\text{T}} } \right)\left( {{\varvec{y}}{\varvec{y}}^{\text{T}} } \right)} \right]^{{{1 \mathord{\left/ {\vphantom {1 2}} \right. \kern-0pt} 2}}} }},$$

where ${\varvec{x}}_{{}}^{{\text{T}}}$ and ${\varvec{y}}_{{}}^{{\text{T}}}$ represent the transpositions of $\user2{x}$ and ${\varvec{y}}$, respectively. The range of SAM value is $[0,1]$. The smaller the value, the higher the similarity.

Similarly, the spectral angle cosine (SAC) method converts the range to $[ - 1,1]$. The higher the SAC value, the higher the similarity between the reference spectrum vector and the test spectrum vector. The SAC is defined as follows:

3 $${\text{SAC}} \left( {{\varvec{x}},{\varvec{y}}} \right) = \frac{{{\varvec{xy}}_{{}}^{\text{T}} }}{{\left[ {\left( {{\varvec{xx}}_{{}}^{\text{T}} } \right)\left( {{\varvec{yy}}_{{}}^{{\text{T}}} } \right)} \right]^{{{1 \mathord{\left/ {\vphantom {1 2}} \right. \kern-0pt} 2}}} }}.$$

The SGA method measures the similarity between spectral gradient vectors, as defined in Eq. (4). It takes into account the tilt of the vector and is robust to geometric distortions and intensity variations. The range of SGA value is $[ - 1,1]$. By converting the range to [0,1], the NSGA method can be defined, as in Eq. (5):

4 $${\text{SGA}} \left( {{\varvec{x}},{\varvec{y}}} \right) = {\text{SAC}} \left( {{\varvec{x^{\prime}}},{\varvec{y^{\prime}}}} \right),$$

5 $${\text{NSGA}} = ({\text{SGA}} + 1)/2,$$

where ${\varvec{x^{\prime}}} = [{\varvec{x}}_{2} - {\varvec{x}}_{1} ,{\varvec{x}}_{3} - {\varvec{x}}_{2} ,...,{\varvec{x}}_{n} - {\varvec{x}}_{n - 1} ]$, ${\varvec{y^{\prime}}} = [{\varvec{y}}_{2} - {\varvec{y}}_{1} ,{\varvec{y}}_{3} - {\varvec{y}}_{2} ,...,{\varvec{y}}_{n} - {\varvec{y}}_{n - 1} ]$. The spectral gradient reflects the slope of the spectral vector and describes the morphological features of the spectral vector. Typically, these changes are associated with the absorption features of the targets, which is one of the essential features of an image.

### Distance-based TD methods

Distance-based TD methods assume that the spectrum is a higher dimensional vector in the Euclidean space, where dimension is the number of spectral bands. The TD problem is transformed into a similarity measurement problem by calculating the distance between two vectors. The smaller the distance, the greater the spectral similarity. Distance-based TD methods include Euclidean distance (ED) method, the normalized Euclidean distance (NED) [15] method, the Mahalanobis distance (MaD) method [16], Chebyshev distance (TcD) method [17], and Hamming distance (HD) method [18], etc.

The ED method is a frequently-used method for distance-based TD. However, the detection results of ED method are largely dependent on the spectral amplitude, and are insensitive to the differences in the spectral shape. To solve this problem, the NED is defined, as in Eq. (6):

6 $${\text{NED}} \left( {{\varvec{x}},{\varvec{y}}} \right) = \left\| {{\varvec{x}} - {\varvec{y}}} \right\|_{{{\text{L}}_{2} }} = \sqrt {\sum\limits_{i = 1}^{n} {\left( {{\tilde{\varvec{x}}}_{i} - {\tilde{\varvec{y}}}_{i} } \right)}^{2} } ,$$

where ${\tilde{\varvec{x}}}_{i} = \frac{{{\varvec{x}}_{i} }}{{\left\| {\varvec{x}} \right\|}}$, ${\varvec{\tilde{y}}}_{i} = \frac{{{\varvec{y}}_{i} }}{{\left\| {\varvec{y}} \right\|}}$. For this method, spectrum vectors are normalized before calculating the Euclidean distance, such that the range of NED values is $[0,1]$. The smaller the NED value, the more similar the two spectral vectors. The robustness is enhanced after normalization.

### Information-based TD methods

Information-based TD methods are based on evaluating the information entropy (IE) characteristics of the spectral vectors. IE describes the uncertainty of the signal and reflects the amount of information.

Mutual information (MI) is used to describe the correlation between two systems, that is, the amount of information contained in both systems. The MI between the test spectrum vector and the reference spectrum vector is defined, as in Eq. (7):

7 $${\text{MI}} ({\varvec{x}},{\varvec{y}}) = \sum\limits_{i = 1}^{n} {\sum\limits_{j = 1}^{n} {p({\varvec{x}}_{i} ,{\varvec{y}}_{j} )\log \frac{{p({\varvec{x}}_{i} ,{\varvec{y}}_{j} )}}{{p({\varvec{x}}_{i} )p({\varvec{y}}_{j} )}}} } ,$$

where $p({\varvec{x}}_{i} ) = \frac{{{\varvec{x}}_{i} }}{{\sum\limits_{i = 1}^{n} {{\varvec{x}}_{i} } }}$, $p({\varvec{y}}_{j} ) = \frac{{{\varvec{y}}_{j} }}{{\sum\limits_{j = 1}^{n} {{\varvec{y}}_{j} } }}$. $p({\varvec{x}}_{i} ,{\varvec{y}}_{j} )$ represents the joint probability density of ${\varvec{x}}_{i}$ and ${\varvec{y}}_{i}$.The larger the value of MI, the larger the amount of duplicated information, thus the greater the correlation between the two spectral vectors and the more similar the spectra.

### Statistics-based TD methods 

Statistics-based TD methods measure the similarity by calculating the correlation between the reference spectrum vector and the test spectrum vector [19]. Representative methods include the normalized correlation coefficient (NCC) and spectral correlation angle (SCA) methods. The NCC and SCA methods are defined as follows:

8 $${\text{NCC}} ({\varvec{x}},{\varvec{y}}) = (R_{xy} + 1)/2,$$

9 $${\text{SCA}} ({\varvec{x}},{\varvec{y}}) = \arccos {\text{NCC}} ({\varvec{x}},{\varvec{y}}),$$

10 $$R_{xy} = \frac{{{\text{cov}} ({\varvec{x}},{\varvec{y}})}}{{\sigma_{x} \sigma_{y} }} = \frac{{\sum {({\varvec{x}}_{i} - \overline{x})({\varvec{y}}_{i} - \overline{y})} }}{{\sqrt {\sum {({\varvec{x}}_{i} - \overline{x})^{2} \cdot \sum {({\varvec{y}}_{i} - \overline{y})^{2} } } } }},$$

11 $$\begin{gathered} \overline{x} = \frac{{\sum {{\varvec{x}}_{i} } }}{n}, \hfill \\ \overline{y} = \frac{{\sum {{\varvec{y}}_{i} } }}{n}, \hfill \\ \end{gathered}$$

where $R_{xy}$ represents the correlation coefficient between $\user2{x}$ and ${\varvec{y}}$. ${\text{cov}} ({\varvec{x}},{\varvec{y}})$ represents the covariance between $\user2{x}$ and ${\varvec{y}}$. $\sigma_{x}$ and $\sigma_{y}$ represent the standard deviations of $\user2{x}$ and ${\varvec{y}}$, respectively. The range of the NCC value is $[0,1]$. The larger the value, the higher the similarity. The range of the SCA value is $[0,\frac{\uppi }{2}]$. The smaller the value, the higher the similarity.

### Constrained energy minimization (CEM) method

The principle of the CEM method is to design a linear filter vector ${\varvec{w}} = [w_{1} ,w_{2} ,...,w_{n} ]^{\text{T}}$ that minimizes the energy of the filtered output of the test hyperspectral image and satisfies Eq. (12) simultaneously:

12 $${\varvec{w}}^{\text{T}} {\varvec{x}} = \sum\limits_{i = 1}^{n} {w_{i} {\varvec{x}}_{i} } = 1.$$

The output of the test spectrum vector ***y*** after passing through the filter vector is given by Eq. (13):

13 $$z = {\varvec{w}}^{\text{T}} {\varvec{y}} = \sum\limits_{i = 1}^{n} {w_{i} {\varvec{y}}_{i} } .$$

Therefore, the average output energy of all pixels in the test hyperspectral image is given by Eq. (14):

14 $$\frac{1}{N}\left[ {\sum\limits_{j = 1}^{N} {z_{j}^{2} } } \right] = {\varvec{w}}^{\text{T}} (\frac{1}{N}\sum\limits_{j = 1}^{N} {{\varvec{y}}_{j} {\varvec{y}}_{j}^{\text{T}} } ){\varvec{w}} = {\varvec{w}}^{\text{T}} {{R}}\user2{w,}$$

where ${{R}} = \frac{1}{N}\sum\limits_{j = 1}^{N} {{\varvec{y}}_{j} {\varvec{y}}_{j}^{\text{T}} }$ represents the autocorrelation matrix of all pixels in the hyperspectral image. $N$ represents the number of pixels in the hyperspectral image. $z_{j}$ represents the output of the *j*-th pixel in the hyperspectral image. Then the filter design problem is transformed into a minimum value problem satisfying both Eq. (12) and Eq. (15):

15 $$\mathop {\min }\limits_{{\varvec{w}}} ({\varvec{w}}^{\text{T}} {{R}}{\varvec{w}}) = \mathop {\min }\limits_{{\varvec{w}}} ({\varvec{w}}^{\text{T}} \frac{1}{N}\sum\limits_{j = 1}^{N} {{\varvec{y}}_{j} {\varvec{y}}_{j}^{\text{T}} } {\varvec{w}}).$$

Equation (15) can be solved by the Lagrange multiplier method:

16 $${\varvec{w}} = \frac{{{{R}}^{ - 1} {\varvec{x}}}}{{{\varvec{x}}^{\text{T}} {{R}}^{ - 1} {\varvec{x}}}}.$$

The filter is applied to each pixel to achieve TD.

17 $${\text{CEM}} ({\varvec{y}}) = {\varvec{w}}^{\text{T}} {\varvec{y}} = \frac{{{\varvec{y}}^{\text{T}} {{R}}^{ - 1} {\varvec{x}}}}{{{\varvec{x}}^{\text{T}} {{R}}^{ - 1} {\varvec{x}}}}.$$

By analyzing the above traditional TD methods, we find that the projection-based and statistics-based TD methods can overcome the influence of spectral amplitude change induced by the change of illumination intensity, terrain, shadow, or other factors, because it is invariant to multiplicative factors. Theoretically, these methods are more suitable for land-based imaging.

The following equation shows a comparison of the three methods based on projection and statistics: the NSGA, SAC, and SCA methods:

18 $$\begin{gathered} \quad \, \, {\text{NSGA}} \left( {k{\varvec{y}} + {\varvec{M}},{\varvec{x}}} \right) \ne {\text{NSGA}} \left( {{\varvec{x}},{\varvec{y}}} \right), \\ {\text{SAC}} \left( {k{\varvec{y}} + {\varvec{M}},{\varvec{x}}} \right) \ne {\text{SAC}} \left( {{\varvec{x}},{\varvec{y}}} \right), \\ {\text{SCA}} \left( {k{\varvec{y}} + {\varvec{M}},{\varvec{x}}} \right) = {\text{SCA}} \left( {{\varvec{x}},{\varvec{y}}} \right), \\ \end{gathered}$$

where ${\varvec{M}}$ represents an arbitrary constant vector. Compared to the SAC and NSGA methods, the SCA method is invariant to both multiplicative factors and additive factors. It can more effectively suppress the influence of imaging conditions, such as shadowing and illumination, on similarity measurement. Therefore, it is more suitable for measuring the spectral similarity in land-based conditions. However, this approach has some drawbacks. It can only reflect the overall similarity features but cannot exploit the local features of the spectral vectors. In land-based imaging, the uncertainties are different in different spectral bands. Therefore, it is still difficult to effectively reduce the effect of spectral uncertainties on TD. In this paper, we propose to classify the spectral bands and assign different weights to exploit the local features of the spectral vectors.

## Weighted spectral correlation angle (WSCA) method

In the proposed weighted spectral correlation angle (WSCA) method, the full bands are divided into feature and common bands, and appropriate weights are assigned to exploit local features. The feature bands refer to the bands with large uncertainties under different imaging conditions. Common bands are those where these uncertainties are small. Assume that the amount of feature and common bands are *N*_*a*_ and *N*_*b*_ respectively. So $N_{{{a}}} + N_{{{b}}} = N$. ***x*** and ***y*** ub the feature bands are represented by ***x***_*a*_ and ***y***_*a*_, respectively. While ***x*** and ***y*** in the common bands of are represented by ***x***_*b*_ and ***y***_*b*_, respectively. The weight *k* is greater than 0. The WSCA method can be expressed as follows:

19 $${\text{WSCA}} ({\varvec{x}},{\varvec{y}}) = \arccos [(R_{xy}^{\prime} + 1)/2],$$

where

20 $$R_{{xy}}^{'} = \frac{{\sum {(\user2{x}_{i} - \bar{x})(\user2{y}_{i} - \bar{y})} + k\sum\nolimits_{{\text{b} _{j} = 1}}^{{N_{\text{b} } }} {(\user2{x}_{{\text{b} _{j} }} - \bar{x})(\user2{y}_{{\text{b} _{j} }} - \bar{y})} }}{{\sqrt {[\sum {(\user2{x}_{i} - \bar{x})^{2} + k\sum\nolimits_{{\text{b} _{j} = 1}}^{{N_{\text{b} } }} {(\user2{x}_{{\text{b} _{j} }} - \bar{x})^{2} } ][\sum {(\user2{y}_{i} - \bar{y})^{2} + k\sum\nolimits_{{\text{b} _{j} = 1}}^{{N_{\text{b} } }} {(\user2{y}_{{\text{b} _{j} }} - \bar{y})^{2} } ]} } } }},$$

where $R_{xy}^{\prime}$ represents the weighted correlation coefficient between $\user2{x}$ and ${\varvec{y}}$ in the full bands. It can be seen from Eq. (19) that the value of WSCA is inversely proportional to $R_{xy}^{\prime}$. The larger the $R_{xy}^{\prime}$, the smaller the WSCA value and the more similar the two spectral vectors. $R_{xy}^{\prime}$ can be also written as follows:

21 $$R_{xy}^{\prime} = \frac{{(k + 1)\sum {({\varvec{x}}_{i} - \overline{x})({\varvec{y}}_{i} - \overline{y})} - k\sum\nolimits_{{{a}_{i} = 1}}^{{N_{a} }} {({\varvec{x}}_{{{a}_{i} }} - \overline{x})({\varvec{y}}_{{{a}_{i} }} - \overline{y})} }}{{\sqrt {\left[ {(k + 1)\sum {({\varvec{x}}_{i} - \overline{x})^{2} } - k\sum\nolimits_{{{{a}}_{i} = 1}}^{{N_{a} }} {({\varvec{x}}_{{{a}_{i} }} - \overline{x})^{2} } } \right]\cdot\left[ { {(k + 1)} \sum {({\varvec{y}}_{i} - \overline{y})^{2} } - k\sum\nolimits_{{{a}_{i} = 1}}^{{N_{a} }} {({\varvec{y}}_{{{a}_{i} }} - \overline{y})^{2} } } \right]} }}.$$

Set $$\sum {({\varvec{x}}_{i} - \overline{x})({\varvec{y}}_{i} - \overline{y})} = f$$, $$\sqrt {\sum {({\varvec{x}}_{i} - \overline{x})^{2} } } = F_{x}$$, $$\sqrt {\sum {({\varvec{y}}_{i} - \overline{y})^{2} } } = F_{y}$$, then:

22 $$\begin{gathered} \eta =  R_{xy}^{\prime} - R_{xy} = \frac{{(k + 1)f - k\sum\nolimits_{{{{a}}_{i} = 1}}^{{N_{{a}} }} {({\varvec{x}}_{{{{a}}_{i} }} - \overline{x})({\varvec{y}}_{{{{a}}_{i} }} - \overline{y})} }}{{\sqrt {\left[ {(k + 1)F_{x}^{2} - k\sum\nolimits_{{{{a}}_{i} = 1}}^{{N_{{a}} }} {({\varvec{x}}_{{{{a}}_{i} }} - \overline{x})^{2} } } \right]\left[ {(k + 1)F_{y}^{2} - k\sum\nolimits_{{{{a}}_{i} = 1}}^{{N_{{a}} }} {({\varvec{y}}_{{{{a}}_{i} }} - \overline{y})^{2} } } \right]} }} - \frac{f}{{F_{x} \cdot F_{y} }} \cdot \hfill \\ \qquad \frac{{\sqrt {(k + 1)^{2} F_{x}^{2} F_{y}^{2} - k(k + 1)F_{x}^{2} \sum\nolimits_{{{{a}}_{i} = 1}}^{{N_{{a}} }} {({\varvec{y}}_{{{{a}}_{i} }} - \overline{y})^{2} } - k(k + 1)F_{y}^{2} \sum\nolimits_{{{{a}}_{i} = 1}}^{{N_{a} }} {({\varvec{x}}_{{{{a}}_{i} }} - \overline{x})^{2} } + k^{2} \sum\nolimits_{{{{a}}_{i} = 1}}^{{N_{{a}} }} {({\varvec{y}}_{{{{a}}_{i} }} - \overline{y})^{2} } \cdot \sum\nolimits_{{{{a}}_{i} = 1}}^{{N_{{a}} }} {({\varvec{x}}_{{{{a}}_{i} }} - \overline{\user2{x}})^{2} } } }}{{\sqrt {\left[ {(k + 1)F_{x}^{2} - k\sum\nolimits_{{{{a}}_{i} = 1}}^{{N_{a} }} {({\varvec{x}}_{{{{a}}_{i} }} - \overline{x})^{2} } } \right]\left[ {(k + 1)F_{y}^{2} - k\sum\nolimits_{{{{a}}_{i} = 1}}^{{N_{{a}} }} {({\varvec{y}}_{{{{a}}_{i} }} - \overline{y})^{2} } } \right]} }}. \hfill \\ \end{gathered}$$

Because

23 $$- k(k + 1)F_{x}^{2} \sum\nolimits_{{{{a}}_{i} = 1}}^{{N_{a} }} {({\varvec{y}}_{{{{a}}_{i} }} - \overline{y})^{2} } - k(k + 1)F_{y}^{2} \sum\nolimits_{{{{a}}_{i} = 1}}^{{N_{a} }} {({\varvec{x}}_{{{{a}}_{i} }} - \overline{x})^{2} } \le - 2k(k + 1)F_{x} F_{y} \sqrt {\sum\nolimits_{{{{a}}_{i} = 1}}^{{N_{a} }} {({\varvec{y}}_{{{{a}}_{i} }} - \overline{y})^{2} } \cdot \sum\nolimits_{{{{a}}_{i} = 1}}^{{N_{a} }} {({\varvec{x}}_{{{{a}}_{i} }} - \overline{x})^{2} } } ,$$

it follows that

24 $$\eta \ge \frac{{(k + 1)f - k\sum\nolimits_{{{{a}}_{i} = 1}}^{{N_{{a}} }} {({\varvec{x}}_{{{{a}}_{i} }} - \overline{x})({\varvec{y}}_{{{{a}}_{i} }} - \overline{y})} }}{{\sqrt {\left[ {(k + 1)F_{x}^{2} - k\sum\nolimits_{{{{a}}_{i} = 1}}^{{N_{{a}} }} {({\varvec{x}}_{{{{a}}_{i} }} - \overline{x})^{2} } } \right]\left[ {(k + 1)F_{y}^{2} - k\sum\nolimits_{{{{a}}_{i} = 1}}^{{N_{{a}} }} {({\varvec{y}}_{{{{a}}_{i} }} - \overline{y})^{2} } } \right]} }} - \frac{{\frac{f}{{F_{x} \cdot F_{y} }}\left| {(k + 1)F_{x} F_{y} - k\sqrt {\sum\nolimits_{{{{a}}_{i} = 1}}^{{N_{a} }} {({\varvec{y}}_{{{{a}}_{i} }} - \overline{y})^{2} } \cdot \sum\nolimits_{{{{a}}_{i} = 1}}^{{N_{a} }} {({\varvec{x}}_{{{{a}}_{i} }} - \overline{x})^{2} } } } \right|}}{{\sqrt {\left[ {(k + 1)F_{x}^{2} - k\sum\nolimits_{{{{a}}_{i} = 1}}^{{N_{{a}} }} {({\varvec{x}}_{{{{a}}_{i} }} - \overline{x})^{2} } } \right]\left[ {(k + 1)F_{y}^{2} - k\sum\nolimits_{{{{a}}_{i} = 1}}^{{N_{a} }} {({\varvec{y}}_{{{{a}}_{i} }} - \overline{y})^{2} } } \right]} }}.$$

Because

25 $$(k + 1)F_{x} F_{y} - k\sqrt {\sum\nolimits_{{{{a}}_{i} = 1}}^{{N_{{a}} }} {({\varvec{y}}_{{{{a}}_{i} }} - \overline{y})^{2} } \cdot \sum\nolimits_{{{{a}}_{i} = 1}}^{{N_{{a}} }} {({\varvec{x}}_{{{{a}}_{i} }} - \overline{x})^{2} } } > 0,$$

it follows that

26 $$\eta \ge \frac{{k\sqrt {\sum\nolimits_{{{{a}}_{i} = 1}}^{{N_{{a}} }} {({\varvec{y}}_{{{{a}}_{i} }} - \overline{y})^{2} } \cdot \sum\nolimits_{{{{a}}_{i} = 1}}^{{N_{{a}} }} {({\varvec{x}}_{{{{a}}_{i} }} - \overline{x})^{2} } } \left( {R_{xy} - R_{{xy - N_{{a}} }} } \right)}}{{\sqrt {\left[ {(k + 1)F_{x}^{2} - k\sum\nolimits_{{{{a}}_{i} = 1}}^{{N_{{a}} }} {({\varvec{x}}_{{{{a}}_{i} }} - \overline{x})^{2} } } \right]\left[ {(k + 1)F_{y}^{2} - k\sum\nolimits_{{{{a}}_{i} = 1}}^{{N_{{a}} }} {({\varvec{y}}_{{{{a}}_{i} }} - \overline{y})^{2} } } \right]} }},$$

where $R_{{xy - N_{{{a}}} }} = \frac{{\sum\nolimits_{{{{a}}_{i} = 1}}^{{N_{{a}} }} {({\varvec{x}}_{{{{a}}_{i} }} - \overline{x})({\varvec{y}}_{{a_{i} }} - \overline{y})} }}{{\sqrt {\sum\nolimits_{{a_{i} = 1}}^{{N_{{a}} }} {({\varvec{y}}_{{a_{i} }} - \overline{y})^{2} } \cdot \sum\nolimits_{{a_{i} = 1}}^{{N_{{a}} }} {({\varvec{x}}_{{a_{i} }} - \overline{x})^{2} } } }}$ represents the correlation coefficients between $\user2{x}$ and ${\varvec{y}}$ for the feature bands and was called feature correlation coefficients for simplicity. It can be seen from the equation above that when the feature bands are properly selected, such that $R_{{xy - N_{{{a}}} }} < R_{xy}$ is satisfied, then $\eta > 0$ and ${\text{WSCA}} ({\varvec{x}},{\varvec{y}}) < {\text{SCA}} ({\varvec{x}},{\varvec{y}})$. The similarity between the reference spectrum and the test spectrum is increased and the similarity increases with increase of *k*. Therefore, it is only necessary to select the feature bands that $R_{{xy - N_{{{a}}} }} < R_{xy}$ is satisfied so as to increase the similarity between the reference spectrum and the test spectrum, thus decreasing the influence of spectral uncertainties on TD in land-based conditions.

## Experiment and analysis

To verify the performance of the WSCA method in reducing the influence of spectral uncertainties on TD, two sets of experiments were conducted. The same scene is imaged by an imaging spectrometer under different imaging conditions to obtain different groups of hyperspectral data. The imaging conditions include weather conditions, atmospheric conditions, light conditions, time conditions, zenith and other azimuth angle conditions that affect imaging result, which are different due to different imaging times.. In Experiment 1, only the spectral uncertainties caused by different imaging conditions are taken into account and the performance of the WSCA method in reducing the influence of spectral uncertainties are verified. In Experiment 2, imaging condition is kept unchanged to verify the performance of the WSCA method in reducing the influence of spectral uncertainties caused by an uneven spatial distribution.

The experimental data were obtained by an HSI-300 spectrometer. The spectral resolution (band interval) of HSI-300 spectrometer was 4 nm, and the bandwidth of each band was 2.3 nm. The band range of the HSI-300 spectrometer was 449–801 nm. The experiment took place in Shijiazhuang, Hebei, China. The geographical coordinates were 38° 27′ N and 114° 30′ E. The date was June 1, 2023. The weather was clear during the experiment. Experiments were conducted at 10:00, 10:30, 11:00, 11:30, 12:00, 12:30, 13:00, 13:30, 14:00, and 14:30, and ten groups of hyperspectral data were obtained. The imaging conditions are listed in Table 1. Each group of hyperspectral data had $712 \times 1002$ pixels and 89 spectral bands.

**Table 1:** Imaging conditions

Group	Time	Solar zenith angle/(˚ )	View zenith angle/(˚ )	Relative azimuth angle/(˚ )
1	10:00	34.5	62	148.5
2	10:30	29.1	62	139.2
3	11:00	24.2	62	130.1
4	11:30	20.0	62	107.6
5	12:00	17.2	62	85.3
6	12:30	16.6	62	66.4
7	13:00	18.5	62	35.5
8	13:30	22.1	62	20.2
9	14:00	26.7	62	13.7
10	14:30	32.0	62	6.1

**Fig. 1:**
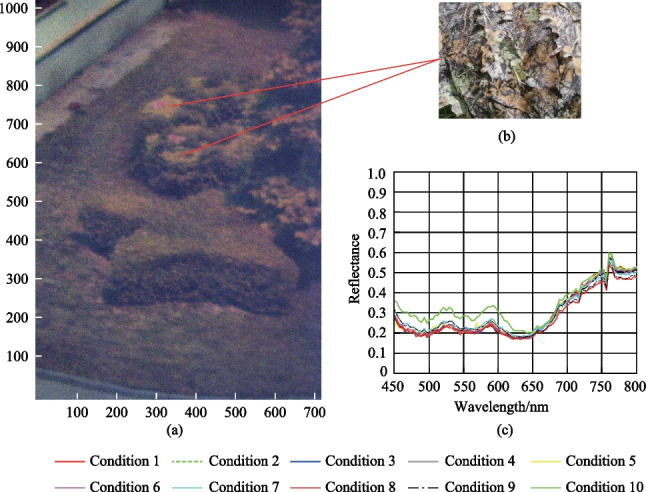
Distribution and spectral curves of the targets. **a** Pseudo-color image of experimental data. **b** Camouflage clothes. **c** Average spectral curves of the targets for different imaging conditions

The targets included two items of camouflaged clothes as shown in Fig. 1a and b. The background was mainly composed of leaves of sycamore trees, leaves of Chinese ilex, soil, and weeds. The targets were almost integrated into the background, and it was difficult to detect their specific locations with the naked eye. The surface of targets was not uniform. Therefore, the spectra at different spatial positions on the target surface are not completely consistent. The arithmetic average of the spectra at all spatial positions on the target surface were taken as the average spectra of the targets under a specific imaging condition. The average spectra of the targets under the imaging conditions given in Table 1 were denoted as S1, S2, S3, S4, S5, S6, S7, S8, S9, and S10, respectively, and are shown in Fig. 1(c).

By analyzing Fig. 1c, we found that the spectra of the targets varied when the imaging conditions changed. It shows obvious spectral uncertainties, mainly concentrated in the band range of 650–800 nm. For condition 10, changes in range of 450–650 nm are larger, which may be attributed to bidirectional reflection characteristics of ground objects have different effects on the spectra of different bands under different imaging conditions.

### Experiment 1: different imaging conditions are considered

The spectrum S1 of the target under imaging condition 1 was taken as the reference spectrum, and the spectra under other imaging conditions were taken as the test spectra. The SAC, NED, SCA, CEM, MI and WSCA methods were used to detect the target under each imaging condition, and the detection results were analyzed. From Eq. 9, detection results with SCA and NCC methods were consistent, as SCA is the arccosine of NCC. Therefore, the NCC method could be used to replace the SCA method in the analysis. The value ranges of the SAC, NED, NCC, CEM, and MI methods were $[0,1]$. The value range of the WSCA method was $\left[ {0,\frac{{\uppi }}{2}} \right]$. To ensure the comparability of the detection results, the detection results of the WSCA method need to be normalized to [0, 1]. Actually, we use the results of $(R_{xy}^{\prime} + 1)/2$ to evaluate the detection results of the WSCA method.

First, the correlation coefficients, feature correlation coefficients, and weighted correlation coefficients of the WSCA method were analyzed. The correlation coefficients between the test spectrum and the reference spectrum under each imaging condition are shown in Table 2.

**Table 2:** Correlation coefficients between the test spectra S1–S10 and the reference spectrum S1

*R*	S1	S2	S3	S4	S5	S6	S7	S8	S9	S10
S1	1	0.9987	0.9987	0.9984	0.9959	0.9982	0.9947	0.9968	0.9922	0.9701

When using the WSCA method, the common and feature bands should be selected first such that $R_{{xy - N_{{{a}}} }} < R_{xy}$ is satisfied. In this experiment, the spectral angle cosine $\omega (i)$ of the reference spectrum and test spectrum in the *i-*th band was calculated to select the common and feature bands.

27 $$\omega (i){ = }SAC\left( {\left[ {\underbrace {{\user2{x}(i),\user2{x}(i),...,\user2{x}(i)}}_{K}} \right],\left[ {\user2{y}_{1} (i),\user2{y}_{2} (i),... ,\user2{y}_{K} (i)} \right]} \right),$$

where *K* represents the number of test spectra. According to the fact that the feature bands occupy a small part of the full bands, we selected 10 feature bands with smaller $\omega$ than other bands, and calculated the feature correlation coefficients $R_{{N_{a} }}$. The results are shown in Table 3.

**Table 3:** Feature correlation coefficients between the test spectra S1–S10 and the reference spectrum S1

$R_{{N_{a} }}$	S1	S2	S3	S4	S5	S6	S7	S8	S9	S10
S1	1	0.9552	0.9478	0.9363	0.7522	0.9618	0.9463	0.9337	0.9307	0.9624

Tables 2 and 3 suggest that $R_{{N_{a} }} < R$ is satisfied for the selected feature bands. Therefore, the WSCA method can be used to increase the spectral similarity of targets in all imaging conditions. When *k* is set as 1, 5 and 10, the weighted correlation coefficients are shown in Table 4.

**Table 4:** Weighted correlation coefficients between the test spectra S1–S10 and the reference spectrum S1

$R^{\prime}$		S1	S2	S3	S4	S5	S6	S7	S8	S9	S10
S1	*k* = 1	1	0.9987	0.9988	0.9984	0.9961	0.9982	0.9947	0.9969	0.9923	0.9702
	*k* = 5	1	0.9987	0.9989	0.9984	0.9962	0.9983	0.9948	0.9970	0.9924	0.9702
	*k* = 10	1	0.9988	0.9990	0.9985	0.9965	0.9984	0.9949	0.9971	0.9926	0.9703

Table 4 suggests that the WSCA method can improve the correlation coefficients, or similarity between the test spectrum and the reference spectrum, which means the robustness of TD can be improved. With the increase of the* k* value, the correlation coefficients further increase, thereby further decreasing the influence of the "different spectra same object" effect on TD.

**Fig. 2:**
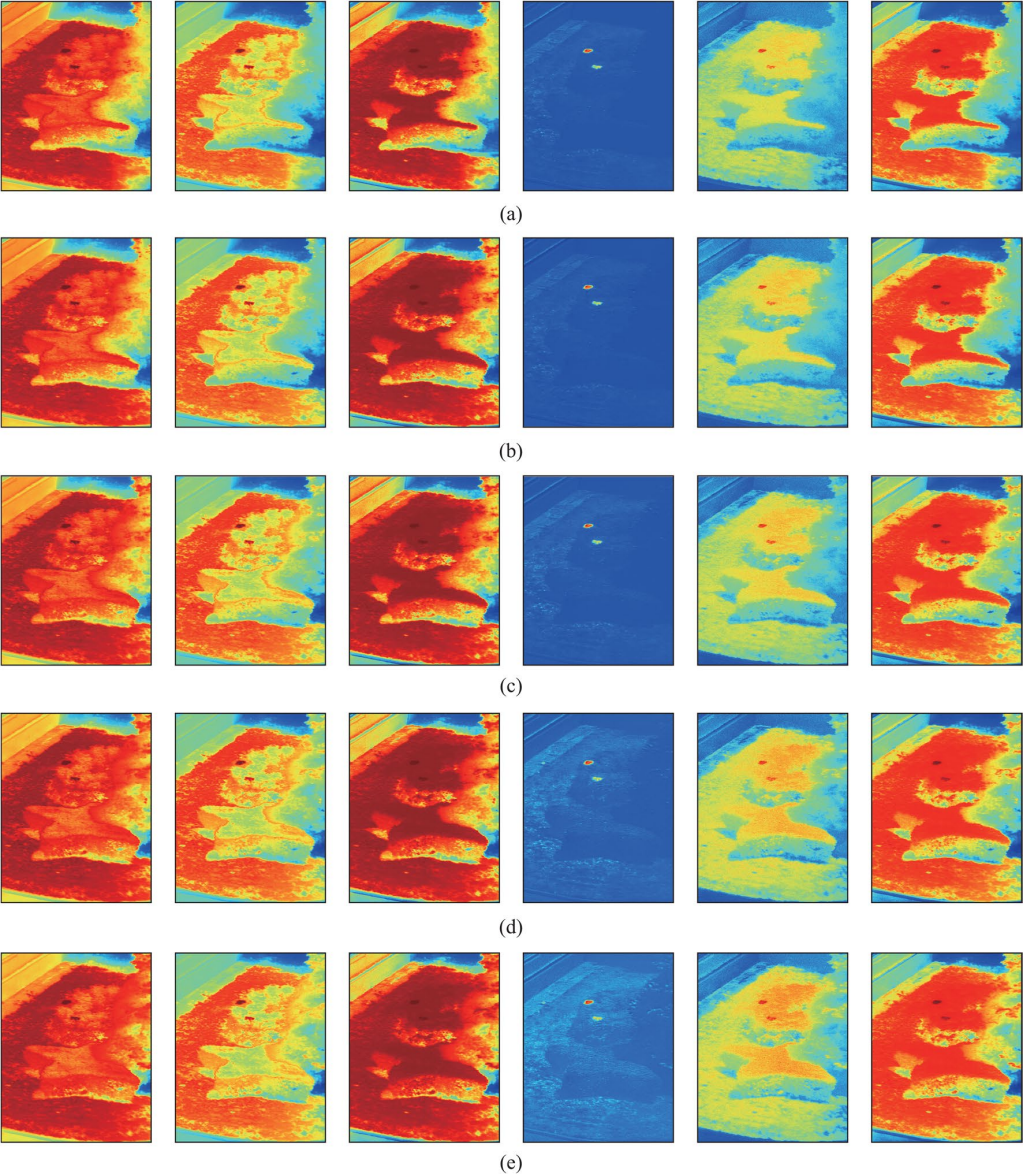

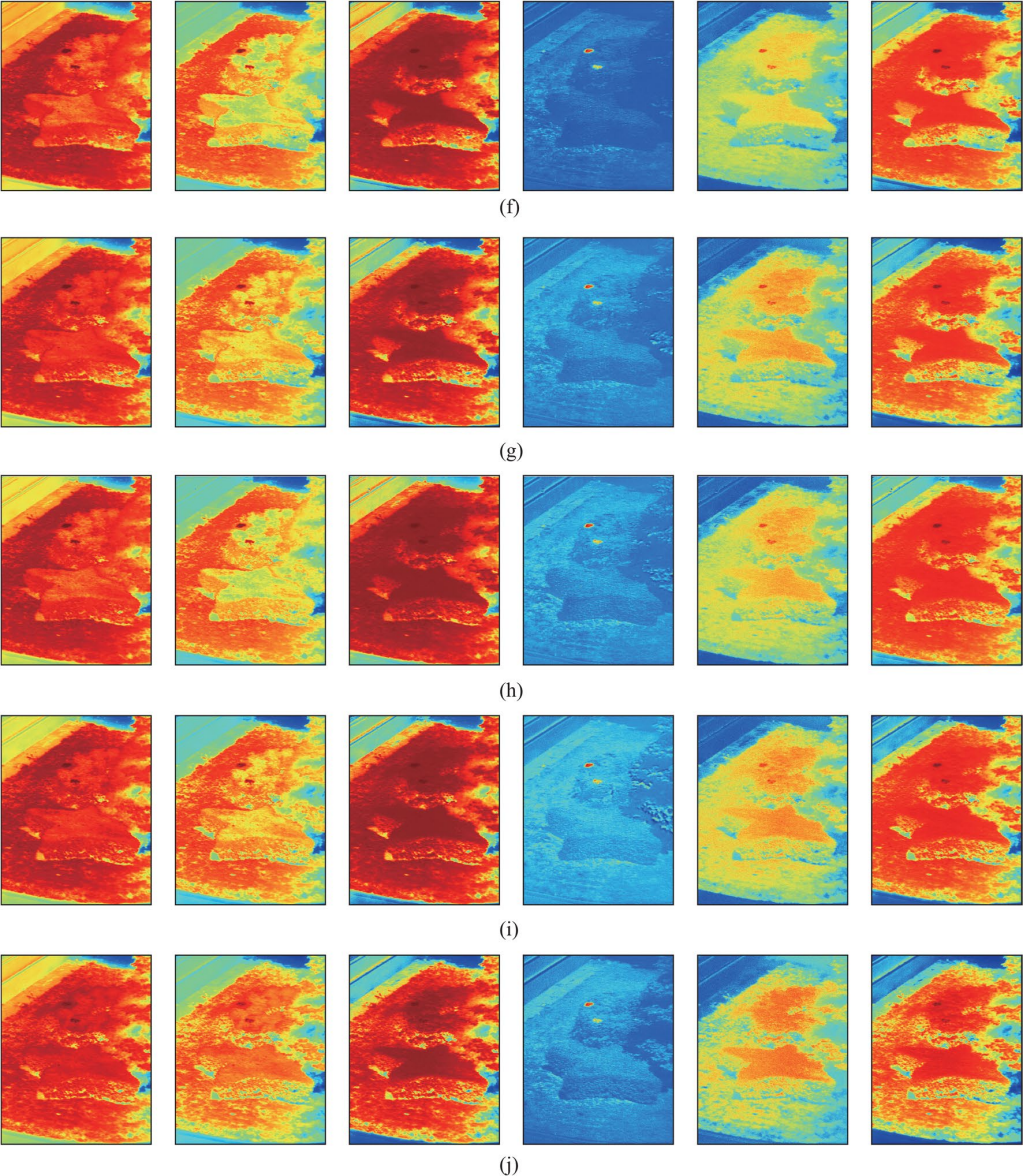
Detection results for each imaging conditions given in Table 1. From left to right, the detection methods are SAC, NED, NCC, CEM, MI, and WSCA. **a** Condition 1. **b** Condition 2. **c** Condition 3. **d** Condition 4. **e** Condition 5. **f** Condition 6. **g** Condition 7. **h** Condition 8. **i** Condition 9. **j** Condition 10

In the following, the targets are detected by using the SAC, NED, NCC, CEM, MI, and WSCA methods under all imaging conditions as specified in Table 1. *k* = 10 is used for the WSCA method. For the NED method, as the test spectrum is more similar to the reference spectrum for smaller NED values, which is different from other methods. 1-NED value is calculated instead for the convenience of comparison. The detection results are shown in Fig. 2.

Figure 2 shows that all methods have good detection results for all of the imaging conditions, and are less affected by imaging conditions. Among them, the CEM method can suppress the pixels whose spectrum is different to the reference spectrum, while enhancing the pixels whose spectrum is similar to the reference spectrum, so the contrast between targets and background is obviously larger than other methods. The targets can be still identified by using the SAC, NED, NCC and WSCA methods, although the contrast between targets and background is relatively smaller. The detection results with MI method are poor for condition 10, indicating that the detection stability of this method is poor.

Figure 2 is a qualitative analysis of the detection results. In fact, a quantitative analysis of the detection results is more convincing. The receiver operating characteristic (ROC) curve [20] and area under the curve (AUC) are used to measure the detection results. The ROC curve reflects the relationship between the probability of detection $P_{{\text{d}}}$ and the probability of false-alarm $P_{{\text{f}}}$ based on a common threshold $\tau$ [21]. Multiple pairs of $P_{{\text{d}}}$ and $P_{{\text{f}}}$ values can be obtained as the threshold changes. $P_{{\text{d}}}$ and $P_{{\text{f}}}$ are defined, as in Eq. (28):

28 $$\left\{ \begin{gathered} P_{{\text{d}}} = \frac{{N_{{\text{d}}} }}{{N_{{\text{t}}} }}, \hfill \\ P_{{\text{f}}} = \frac{{N_{{\text{f}}} }}{{N_{{{\text{tot}}}} - N_{{\text{t}}} }}, \hfill \\ \end{gathered} \right.$$

where $N_{{\text{d}}}$ represents the number of detected true target pixels, that is, the number of pixels that actually belong to the target and are considered as such by the detector. $N_{{\text{t}}}$ represents the total number of target pixels in the image, $N_{{\text{f}}}$ represents the number of false-alarm pixels detected, and $N_{{{\text{tot}}}}$ represents the total number of pixels in the image.

**Fig. 3:**
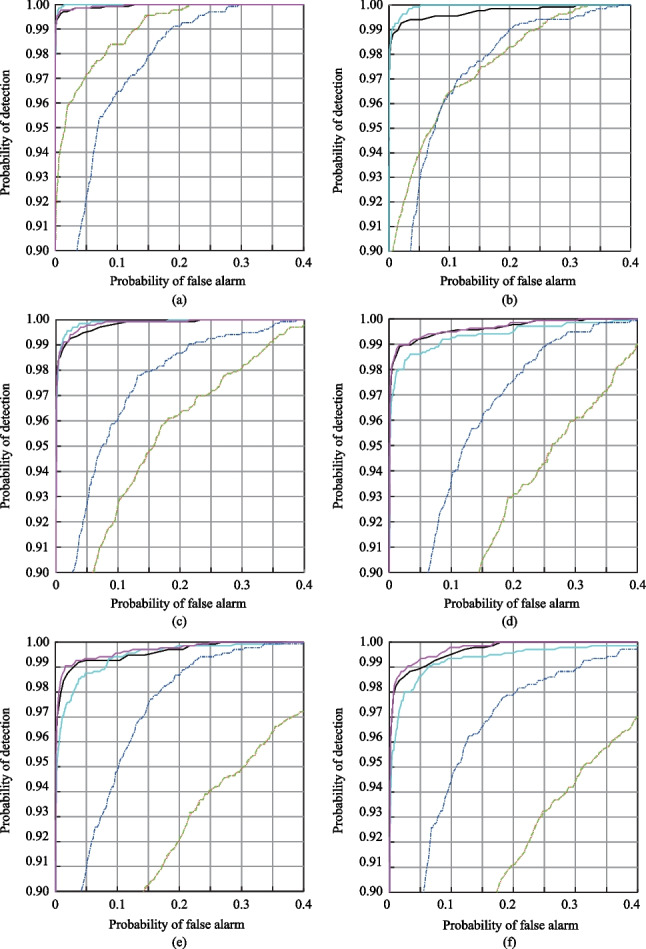

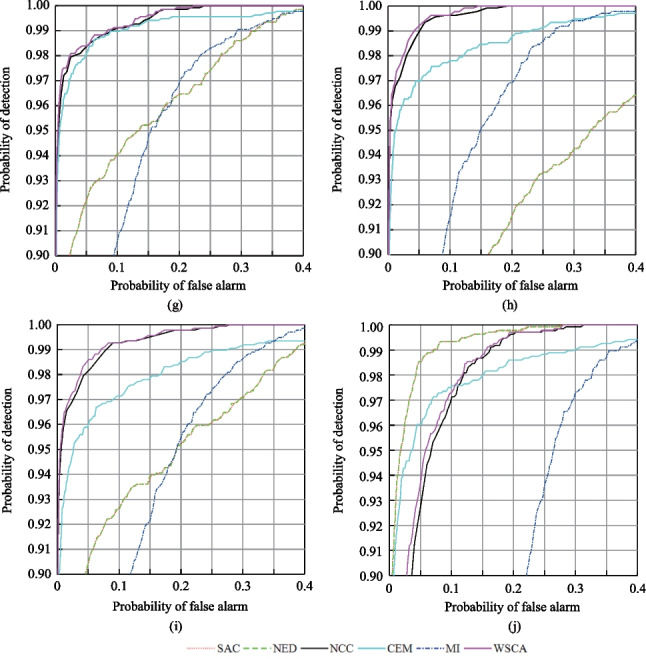
ROC curves for all of the considered methods under different imaging conditions. **a** Condition 1. **b** Condition 2. **c** Condition 3. **d** Condition 4. **e** Condition 5. **f** Condition 6. **g** Condition 7. **h** Condition 8. **i** Condition 9. **j** Condition 10

In fact, the ROC curve is not continuous, but consists of some discrete points. Each discrete point represents the probability of false-alarm and the probability of detectioncorresponding to a threshold. The AUC value is the area under the ROC curve, and quantitatively describes the degree of inclination of the ROC curve to the upper left. The greater the ROC curve is bent to the upper left, the larger the AUC value is, the better the detection method are, and the higher the detection reliability is. The smaller the AUC value is, the worse the detection results are, and the lower the detection reliability is. Figure 3 shows the ROC curves for all of the considered detection methods and imaging conditions, where the step of the threshold is set to 0.001. The AUC values for all of the methods are summarized in Table 5.

**Table 5:** AUC values for all of the considered methods

AUC	SAC	NED	NCC	CEM	MI	WSCA
Condition 1	0.9959	0.9959	0.9998	0.9999	0.9878	0.9998
Condition 2	0.9899	0.9899	0.9989	0.9997	0.9868	0.9992
Condition 3	0.9796	0.9796	0.9992	0.9995	0.9875	0.9994
Condition 4	0.9645	0.9645	0.9985	0.9974	0.9795	0.9988
Condition 5	0.9616	0.9616	0.9983	0.9976	0.9843	0.9986
Condition 6	0.9565	0.9565	0.9985	0.9967	0.9819	0.9988
Condition 7	0.9834	0.9834	0.9974	0.9959	0.9698	0.9976
Condition 8	0.9578	0.9578	0.9981	0.9932	0.9734	0.9984
Condition 9	0.9776	0.9776	0.9970	0.9904	0.9618	0.9973
Condition10	0.9965	0.9965	0.9891	0.9897	0.8959	0.9903
Mean	0.9763	0.9763	0.9975	0.9960	0.9709	0.9978

Figure 3 and Table 5 show that although the detection results of the SAC and NED methods are different, their ROC curves almost coincide, as their detection principles are essentially similar. In addition, under most imaging conditions, ROC curve for the WSCA method has the largest degree of bending to the upper left, and the AUC values are also the largest and less affected by imaging conditions. The average AUC value is also greater than that of other methods. This indicates that the WSCA method has advantages of stability and good detection performance under all of the imaging conditions, and can effectively reduce the influence of “same object different spectrum” effect on TD. In Figs. 2, 3 and Table 5, the *k* value is set to 30, and the *k* value can also be increased to improve the detection performance of the WSCA method more significantly.

### Experiment 2: uneven spatial distribution of targets is considered

**Fig. 4:**
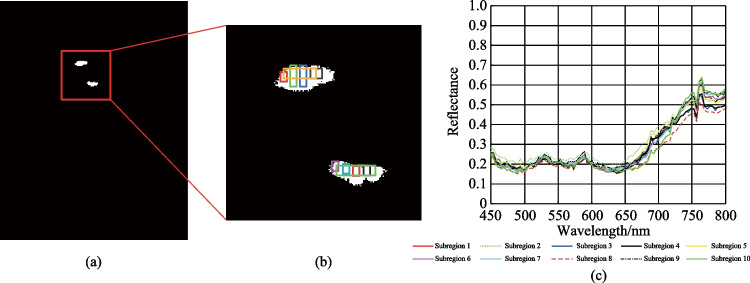
Locations and spectra of the subregions of the targets. **a** Real locations of the targets. **b** Locations of the subregions as marked by rectangle frame of different colors. **c** Average spectra of the targets in each subregion

In this experiment, we assess the performance of the WSCA method in reducing the “same object different spectrum” effect that is caused by uneven spatial distribution. Without loss of generality, the data obtained under imaging condition 6 as specified in Table 1 is used for the analysis. The targets are divided into 10 subregions, and their locations and corresponding spectra are shown in Fig. 4.

Spectrum S6 of the targets under imaging condition 6 is taken as the reference spectrum. The spectra of the subregions under imaging condition 6 are taken as the test spectra. The SAC, NED, SCA, CEM, MI, and WSCA methods are used to detect the target, and the detection results are then analyzed.

First, the correlation coefficients, correlation coefficients in the feature bands, and weighted correlation coefficients of the WSCA method are calculated. The spectrum of each subregion is denoted as R1, R2, R3, R4, R5, R6, R7, R8, R9, and R10, respectively. The correlation coefficients between the reference spectrum S6 and the test spectra R1–R10 are shown in Table 6.

**Table 6:** Correlation coefficients between the test spectra R1–R10 and the reference spectrum S6

*R*	R1	R2	R3	R4	R5	R6	R7	R8	R9	R10
S6	0.9992	0.9987	0.9994	0.9995	0.9993	0.9914	0.9905	0.9872	0.9918	0.9901

Then the spectral angle cosine value is used to choose 10 feature bands, and the feature correlation coefficients are calculated. The results are shown in Table 7.

**Table 7:** Feature correlation Coefficients between the test spectra R1–R10 and the reference spectrum S6

$R_{{N_{a} }}$	R1	R2	R3	R4	R5	R6	R7	R8	R9	R10
S6	0.9692	0.9439	0.9709	0.9913	0.9798	0.9059	0.9659	0.9852	0.9369	0.9264

Table 7 indicates $R_{{N_{{{a}}} }} < R$ is satisfied for the selected feature bands. Therefore, the WSCA method can be used to increase the spectral similarity of the target in all subregions. The weighted correlation coefficients for *k* = 1, 5, 10, respectively, are shown in Table 8.

**Table 8:** Weight correlation coefficients between the test spectra R1–R10 and the reference spectrum S6

$R^{\prime}$		R1	R2	R3	R4	R5	R6	R7	R8	R9	R10
S6	*k* = 1	0.9992	0.9987	0.9994	0.9995	0.9993	0.9914	0.9905	0.9872	0.9918	0.9901
	*k* = 5	0.9993	0.9987	0.9994	0.9995	0.9994	0.9914	0.9905	0.9872	0.9918	0.9901
	*k* = 10	0.9994	0.9988	0.9995	0.9995	0.9994	0.9915	0.9906	0.9873	0.9919	0.9902

**Fig. 5:**
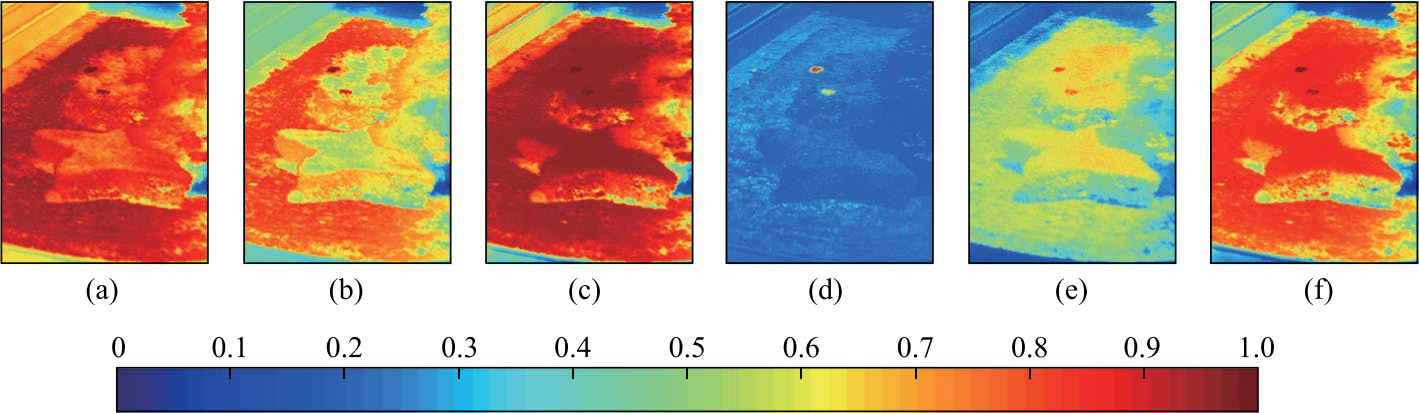
Detection results by using **a** SAC, **b** NED, **c** NCC, **d** CEM, **e** MI, and **f** WSCA methods

**Fig. 6:**
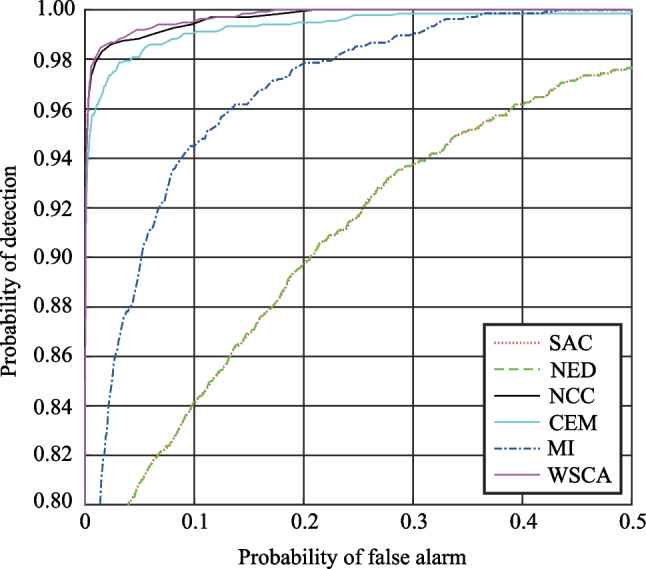
ROC curves of the detection results by using different methods

Table 8 shows that the WSCA method can improve the similarity between the test spectra and the reference spectrum. With the increase of the* k* value, the correlation coefficients increase further, thereby decreasing the influence of “different spectrum same object” on TD. In the following, the targets are detected by using the SAC, NED, NCC, CEM, MI, and WSCA methods. The detection results are shown in Fig. 5. The ROC curves are shown in Fig. 6. The AUC values are shown in Table 9.

**Table 9:** AUC values for all of the considered methods

	SAC	NED	NCC	CEM	MI	WSCA
AUC	0.9494	0.9495	0.9983	0.9961	0.9817	0.9986

Figure 6 shows that ROC curve of the WSCA method has the largest degree of bending to the upper left, and the AUC value is also the largest. This indicates that the WSCA method has a good detection effect and can effectively reduce the influence of “same object different spectrum” on target detection caused by uneven spatial distribution of targets. In Figs. 5 and 6, the *k* value is set to 30, and the *k* value can also be increased to improve the detection effect more significantly. In theory, there is no upper limit to the *k* value, but when the *k* value reaches a certain size, it has little effect on the calculation result.

## Conclusion

In land-based imaging conditions, the spectral uncertainties of ground objects are obvious and are manifested as the so-called “same object different spectrum” effect. This effect has adverse effects on TD and can cause serious missed detections or false-alarms. To solve this problem, this study proposes a WSCA method and compares it with other TD method. WSCA is expected to reduce the influence of spectral uncertainties on TD and improves the TD result. Two groups of experiments are conducted to verify the effectiveness of this method. It is shown that WSCA method significantly improves the probability of detection and reduces the probability of false-alarm, thus enhancing the TD performance. This work provides an effective method for TD for land-based imaging.

## Data Availability

Data underlying the results presented in this paper are not publicly available at this time but may be obtained from the authors upon reasonable request.
